# 2304: Identifying optimal multiple sclerosis (MS)-specific atrophy markers as primary endpoint for Phase II s in progressive MS

**DOI:** 10.1017/cts.2017.127

**Published:** 2018-05-10

**Authors:** Christina Azevedo, Steven Cen, Ling Zheng, Pelletier Amirhossein Jaberzadeh

## Abstract

OBJECTIVES/SPECIFIC AIMS: To identify brain regions with the highest and least variable rate of multiple sclerosis (MS)-specific atrophy using an agnostic approach, and to perform simulation-based sample size calculations for Phase II s using these regions as primary endpoint. METHODS/STUDY POPULATION: In total, 601 subjects (2638 MRI scans) were analyzed; 520 subjects with relapsing forms of MS across the spectrum of disease severity and duration were followed in a single-center prospective cohort study at an academic MS Center between 2005 and 2010 with annual 3 T MRIs and clinical visits for 5 years, including standardized 1 mm^3^ 3D T1-weighted images (3DT1s; 2483 MRIs). Separately, a convenience sample of 81 healthy controls (HC) was recruited from the same center and scanned longitudinally using the same MRI scanner and protocol (155 MRIs). 3DT1s were processed using FreeSurfer’s longitudinal pipeline (software version 5.3). Rates of change in all cortical and subcortical regions (n=119 brain regions) were estimated in MS patients and HC with linear mixed effects models. An effect size was calculated for each region as the difference in change over time between MS patients and HC divided by the standard error of the difference [*d*=β(MS×time)/SE β(MS×time)]. Regions were ranked according to absolute effect size, and the top regions were chosen for simulation-based sample size calculations to estimate the number of subjects needed to achieve 80% power to detect a slowing of MS atrophy down to normal aging, assuming significance levels of 5% and 10%. Ten percent was included because some have advocated for a more relaxed alpha in Phase II s. RESULTS/ANTICIPATED RESULTS: Four regions (putamen, subcortical grey matter, caudate, and thalamus) yielded the smallest sample sizes. At 80% power, ~50 subjects per arm would be needed with putamen or subcortical grey matter volume, or ~80–85 subjects per arm with caudate or thalamic volume as primary endpoint. For the remaining regions, >140 subjects per arm would be needed. A 20%–30% increase in sample size was observed when α=5% was used. DISCUSSION/SIGNIFICANCE OF IMPACT: Using an agnostic approach considering all brain regions and simulation-based sample size calculations specifically designed for longitudinal studies, putaminal, subcortical grey, caudate, and thalamic volumes are sensitive to change over time and yield feasible sample sizes for Phase II studies in MS. Because the effect size estimates incorporate normal aging, these regions represent the most sensitive outcomes for testing therapeutic interventions that target irreversible, MS-specific brain atrophy. The clinical relevance of these regions is our next focus to help inform which of these regions should be favored as primary endpoint.

